# Influence of Ni content on structural, magnetocaloric and electrical properties in manganite La_0.6_Ba_0.2_Sr_0.2_Mn_1−*x*_Ni_*x*_O_3_ (0 ≤ *x* ≤ 0.1) type perovskites

**DOI:** 10.1039/d1ra07059b

**Published:** 2022-02-01

**Authors:** Ahmed Dhahri, J. Laifi, Soumaya Gouadria, M. Elhadi, E. Dhahri, E. K. Hlil

**Affiliations:** a Laboratoire de Physique Appliquée, Faculté des Sciences de Sfax, Université de Sfax BP 1171 3000 Tunisia dhahridhahri14@gmail.com; b Department of Physics, College of Science and Humanities – Dawadmi, Shaqra University Riyadh Saudi Arabia; c Physics Department, College of Science, Jouf University P.O. Box: 2014 Sakaka Saudi Arabia; d Department of Physics, College of Science, Princess Nourah Bint Abdulrahman University P.O. Box 84428 Riyadh 11671 Saudi Arabia; e Univ. Grenoble Alpes, CNRS, Grenoble INP, Institut Néel 38000 Grenoble France

## Abstract

We present a detailed study on the physical properties of La_0.6_Ba_0.2_Sr_0.2_Mn_1−*x*_Ni_*x*_O_3_ samples (*x* = 0.00, 0.05 and 0.1). The ceramics were fabricated using the sol–gel route. Structural refinement, employing the Rietveld method, disclosed a rhombohedral *R*3̄*c* phase. The magnetization *vs.* temperature plots show a paramagnetic–ferromagnetic (PM–FM) transition phase at the *T*_C_ (Curie temperature), which decreases from 354 K to 301 K. From the Arrott diagrams *M*^2^*vs. μ*_0_*H*/*M*, we can conclude the phase transition is of the second order. Based on measurements of the isothermal magnetization around *T*_C_, the magnetocaloric effects (MCEs) have been calculated. The entropy maximum change (−Δ*S*_M_) values are 7.40 J kg^−1^ K^−1^, 5.6 J kg^−1^ K^−1^ and 4.48 J kg^−1^ K^−1^, whereas the relative cooling power (RCP) values are 232 J kg^−1^, 230 J kg^−1^ and 156 J kg^−1^ for *x* = 0.00, 0.05 and 0.10, respectively, under an external field (*μ*_0_*H*) of 5 T. Through these results, the La_0.6_Ba_0.2_Sr_0.2_Mn_1−*x*_Ni_*x*_O_3_ (0 ≤ *x* ≤ 0.1) samples can be suggested for use in magnetic refrigeration technology above room temperature. The electrical resistivity (*ρ*) *vs.* temperature plots exhibit a transition from metallic behavior to semiconductor behavior in the vicinity of *T*_M–SC_. The adiabatic small polaron hopping (ASPH) model is applied in the PM-semiconducting part (*T* > *T*_MS_). Throughout the temperature range, *ρ* is adjusted by the percolation model. This model is based on the phase segregation of FM-metal clusters and PM-insulating regions.

## Abstract 2

We present a detailed study on the physical properties of La_0.6_Ba_0.2_Sr_0.2_Mn_1−*x*_Ni_*x*_O_3_ samples (*x* = 0.00, 0.05 and 0.1).

## Graphical abstract

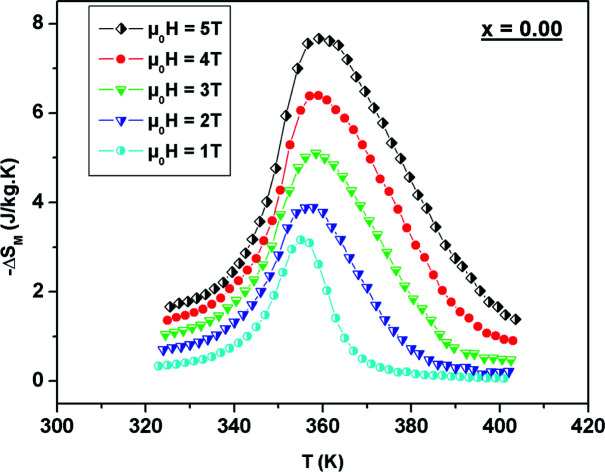

## 1. Introduction

Magnetic refrigeration (MR) technology based on the magnetocaloric effect (MCE) is advancing to become a suitable technology, compared to conventional gas refrigeration,^1–3^ due to a number of advantages.^4^ The MCE is generally characterized by two factors: change in entropy (Δ*S*_M_) and relative cooling power (RCP).

Gadolinium (Gd) is a pure lanthanide element and is the first material that has a high MCE with a Curie temperature (*T*_C_) near room temperature (RT).^5^ Interestingly, further MCE investigations were performed for binary Gd–M compounds, such as Gd_3_SiGe_2_,^6^ which shows a MCE twice that of Gd.^7^ The researchers focused on finding new cheaper materials with larger MCEs. In this context, manganites with perovskite structure have certain advantages over Gd: their elements are not expensive, they are chemically stable, they have high resistivity and they exhibit a good MCE under low magnetic fields.^8,9^ Among these, ABO_3_ compounds are known as perovskite manganites. These materials have a general formula Re_1−*x*_^3+^A_*x*_^2+^(Mn_1−*x*_^3+^Mn_*x*_^4+^)O_3_^2−^ where Re^3+^ is a rare earth element (Nd^3+^, La^3+^, Pr^3+^, Sm^3+^, …) and A^2+^ is an alkaline earth ion (Sr^2+^, Ba^2+^, Ca^2+^).^10,11^ They present some interesting properties, which make them very attractive materials for industrial applications.

The pure stoichiometric lanthanum manganite LaMnO_3_ is antiferromagnetic, insulating at 150 K, and the substitution of the rare earth element by a lower valence ion causes the oxidation of Mn^3+^ into Mn^4+^ to ensure electroneutrality in the material. It is followed by the appearance of macroscopic magnetization, *i.e.* a ferromagnetic coupling between the Mn^3+^ ions (t_2_g_3_e^1^_g_) and the Mn^4+^ ion (t_2_g_3_e^0^_g_). Substitution of La^3+^ by a divalent or monovalent ion can result in a wide Curie temperature range which can vary from 150 K to 375 K. Experimentally, manganites, in particular manganese oxides La_1−*x*_Sr_*x*_MnO_3_ with *x* = 0.3, are well studied systems. They present an FM–PM transition accompanied by a metal–semiconductor transition close to *T*_C_. Several studies have been performed on the magnetocaloric properties of this compound, which exhibits a large change in magnetic entropy, with a narrow range of working temperatures in the vicinity of *T*_C_. In addition, several investigations have been carried out to estimate the substitution effects of the Mn site and these have shown that, for the La_1−*x*_Sr_*x*_MnO_3_ family, even a low rate of substitution at the Mn site would induce a significant change in the properties of magnetotransport.^12–14^ The values of *T*_C_ and Δ*S*_M_ are generally affected by the partial substitution of manganese ions by certain transition metals, for example the In ion,^15,16^ the Al ion,^17^*etc.* On the other hand, the substitution of the rare earth La^3+^ by certain metals lead to a significant change in the magnetic and electrical properties.^18,19^

Indeed, the substitution of the Re site with divalent ions^20^ proves the oxidation of Mn^3+^ to Mn^4+^, which is the origin of the ferromagnetic character.^21,22^ The magnetic coupling between Mn^4+^ and Mn^3+^ is usually governed by the movement of the electron, for example between the two partly filled d layers with a strong Hund’s coupling on site.

The issue of replacing magnetic and non-magnetic ions in the Mn site is very important. For example, the partial substitution of Mn^3+^ ions with Ni^2+^ ions modifies the ratio of the Mn^4+^–O^2^–Mn^3+^ network and leads to a decrease of the double exchange (DE) interactions.^23^ Several studies have been carried out^24–29^ to explain the relationship between the magnetotransport and magnetic properties of Re_1−*x*_A_*x*_MnO_3_ substituted by different elements on the Mn site. Based on the information given in this article, we have carefully discussed the physical properties in La_0.6_Ba_0.2_Sr_0.2_Mn_1−*x*_Ni_*x*_O_3_ (0 ≤ *x* ≤ 0.1) compounds.

## 2. Experimental details

### 2.1 Preparation

The La_0.6_Ba_0.2_Sr_0.2_Mn_1−*x*_Ni_*x*_O_3_ ceramics were prepared using the sol–gel route. High purity precursors La(NO_3_)_3_·6H_2_O, Sr(NO_3_)_2_·6H_2_O, Mn(NO_3_)_2_·4H_2_O, Ba(NO_3_)_2_ and Ni(NO_3_)_2_·6H_2_O were weighed in stoichiometric proportions and then dissolved in distilled water with continuous stirring, whilst on a hot plate. The mixtures were dispersed in solutions containing a complexation agent (citric acid) and a polymerizing agent (ethylene glycol). The citric acid was used as a chelating agent and the ethylene glycol was used as a gelification agent. In order to form a homogenous yellowish gel, the solutions were heated on a hotplate at about 100 °C for 1 h under magnetic stirring. Then, to remove the excess solvent, the temperature was increased to 400 °C and the combustion led to a very fine and very homogeneous powder (black powder). At the end of this process, the calcinated powder was ground and pressed into pellets. The pellets were subjected to sintering at 900 °C for 24 hours in air.

### 2.2 Characterization

X-ray powder diffraction (XRD) was used to examine the structural behavior of the samples. Using a “Panalytical X’Pert Pro” diffractometer, the XRD was conducted through Cu-Kα radiation (*λ*_Cu_ = 1.54056 Å) with a 0.0167° step size and 19 ≤ 2*θ* ≤ 90° angular range. The refinement was analyzed by Rietveld’s program using FULLPROF software (version 0.2-March 1998-LLB-JRC).^30^ A Philips XL30 scanning electron microscope (SEM) and an energy dispersive X-ray (EDX) spectrometer working at 15 kV were used to carry out a morphological study of the compounds. The magnetization measurements were recorded with a BS1 and BS2 magnetometer, which was developed in the Louis Néel laboratory in Grenoble.

## 3. Results and discussion

### 3.1 X-ray analysis

**Fig. 1:**
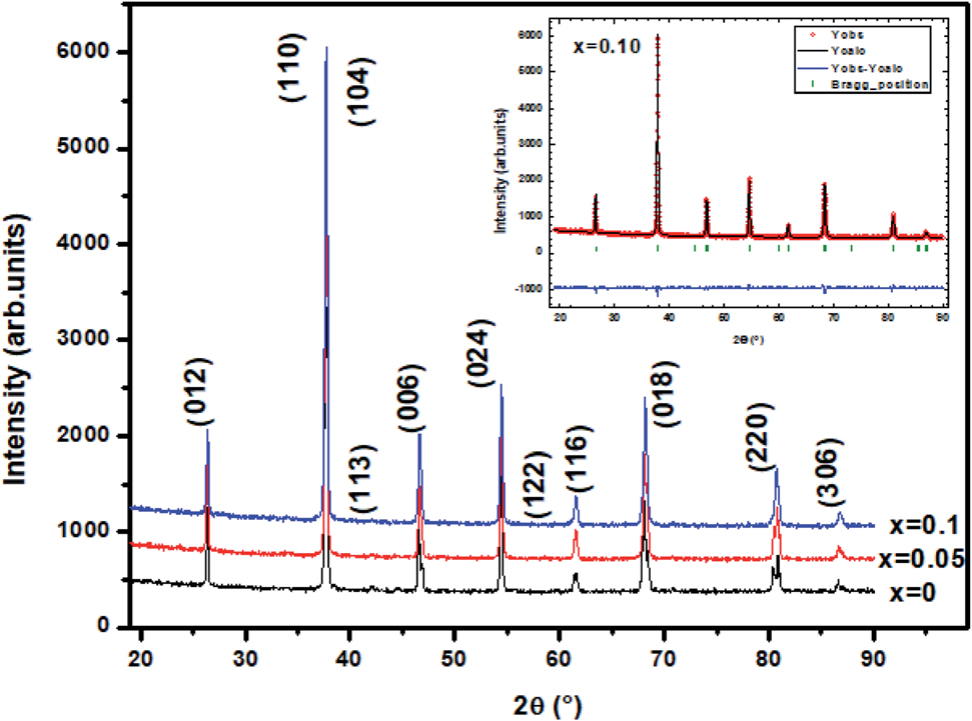
XRD diagrams for solid solutions of the LBSMNO samples. Each of the peaks of these manganites are indexed in the rhombohedral structure. The inset shows the results of the Rietveld’s analysis of sample *x* = 0.10 as an example.

The XRD patterns of the La_0.6_Ba_0.2_Sr_0.2_Mn_1−*x*_Ni_*x*_O_3_ (LBSMNO) samples, recorded at room temperature (RT), are presented in Fig. 1. The analysis of these spectra indicates that all the compounds were successfully prepared with a good crystallinity and a single phase of La_0.6_Ba_0.2_Sr_0.2_Mn_1−*x*_Ni_*x*_O_3_; we have not detected a second phase. In the inset of Fig. 1, we show the crystalline structure of these samples. A good fit agreement between the simulation and the experimental pattern was observed.

The patterns of our samples were indexed in the rhombohedral (*R*3̄*c*) symmetry (no. 167), with (La, Ba, Sr): 6a (0, 0, 0.25), (Mn, Ni): 6b (0, 0, 0) and O: 18 (*x*, 0, 0.25). The different structural parameters are tabulated in Table 1. When the Ni substitution increases, the volume and the lattice parameters decrease. A similar behavior has also been previously observed.^31^

**Table 1:** Structural parameters (X-ray Rietveld refinement) for the LBSMNO samples at RT

	*x*		
	0.00	0.05	0.10
*R*3̄*c* phase			
*a* (Å)	5.4971(4)	5.4824(3)	5.4785(1)
*c* (Å)	13.4713(5)	13.4572(1)	13.4491(3)
*V* (Å^3^)	352.54(1)	350.30(2)	349.58(4)
(O)_Biso_ (Å^2^)	0.85(2)	0.54(3)	2.61(6)
(O)_*x*_	0.4472(1)	0.4526(3)	0.4435(5)
(La, Sr, Ba)_Biso_ (Å^2^)	0.967(1)	0.352(3)	0.853(5)
(Mn, Ni)_Biso_ (Å^2^)	0.368(2)	0.267(7)	1.812(6)

Discrepancy factors			
*R*_wp_ (%)	2.62	3.51	4.11
*R*_p_ (%)	3.22	1.09	4.20
*R*_F_ (%)	5.172	6.532	2.235
*χ* ^2^ (%)	2.42	1.61	1.37

This decrease is explained by the fact that the average ionic radius of the manganese site (0.599 ≤ *r*_Mn+Ni_ ≤ 0.592) decreases, which can be assigned to the formulation of a higher level of Mn^4+^, compared to Mn^3+^.

Taking into account the neutrality of La_0.6_^3+^Ba_0.2_^2+^Sr_0.2_^2+^(Mn_0.6−*y*_^3+^Mn_0.4−*x*+*y*_^4+^)Ni_*x*_^2+^O_3_^2−^ (0 ≤ *x* ≤ 0.1), these results can be justified as follows: whenever the *x* ratio of Ni is increased, the proportion of Mn^3+^ weakens by *y* = 2*x*, while the ratio of Mn^4+^ increases by *x*. In Table 2, we have listed the (Mn/Ni)–O–(Mn/Ni) bond angles (*θ*_(Mn/Ni)–O–(Mn/Ni)_) and the (Mn/Ni)–O bond lengths (*d*_(Mn/Ni)–O_). It is noted that *θ*_(Mn/Ni)–O–(Mn/Ni)_ decreases linearly with the increase of *x*, whereas *d*_(Mn/Ni)–O_ increases, leading to a tilting of the BO_6_ octahedrons.

**Table 2:** Values for the average distance, angle, ionic radius 〈*r*_B_〉, one-electron band-width *W*, tolerance factor *t*_G_, distortion factor *D* and particle size of the LBSMNO samples

*x*	0.00	0.05	0.10
*θ*_(Mn/Ni)–O–(Mn/Ni)_ (°)	166.2(1)	164.98(9)	162.82(6)
*d*_(Mn/Ni)–O_ (Å)	1.9571(1)	1.9587(2)	1.9633(1)
〈*r*_B_〉 (Å)	0.645	0.647	0.649
*t*_G_	0.974	0.973	0.972
*D*	0.286	0.285	0.283
*W* (10^−2^) (arb. units)	4.73	4.71	4.66
*D*_SC_ (nm)	55	50	49
〈*D*_SEM_〉 ± *σ*_D_ (μm)	1.33 ± 0.44	0.95 ± 0.38	0.79 ± 0.31
*D*_WH_ (nm)	130	121	118
*p* (%)	7.26	7.32	7.35

The perovskite structure can be distorted from the ideal cubic structure, which greatly affects the properties. These distortions are principally given by the relationship between the ionic radius of the cations, defined by the tolerance factor *t*_G_:^32^1
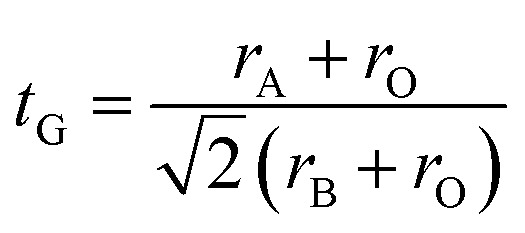
where *r*_A_ is the radius of the A site ions, *r*_B_ is that of the B site ions and *r*_O_ is the radius of the oxygen ions, which are found in the tables of Shannon.^33^ Generally, a perovskite exhibits a cubic structure if *t*_G_ is equal to 1 and it undergoes distortions if *t*_G_ deviates from 1.^34^

In our work, the tolerance factor *t*_G_ decreases with the increase of Ni (Table 2).

The rate of rhombohedric deformation *D*% can be calculated employing the expression: 
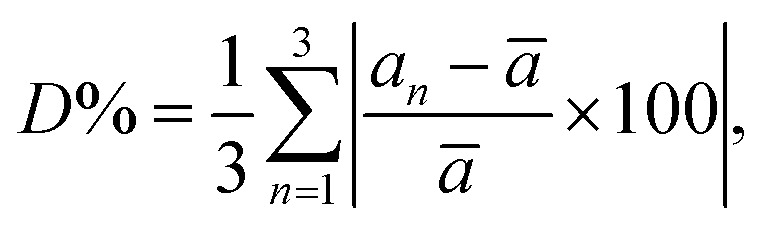
 where 
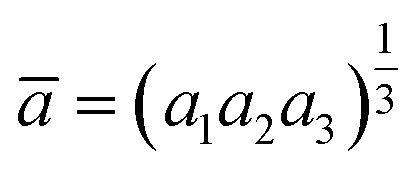
, *a*_1_ = *a*_2_ = *a* and 
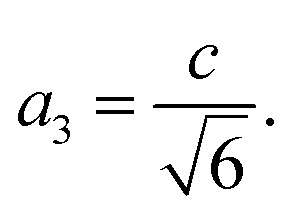
 ^35,36^

From Table 2, the value of *D* decreases with the decrease in mean *r*_Mn+Ni_.

On the other hand, the average crystallite size *D*_SC_ can be calculated using Scherrer’s relationship:^37^2
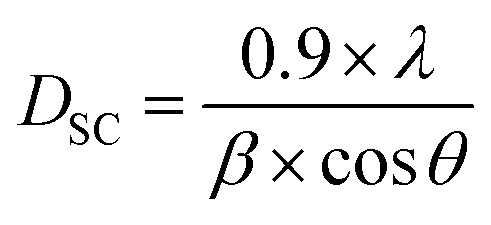
Here, *β* is the full width at half maximum (FWHM) of the peak (104), *λ* represents the wavelength of the Cu-Kα radiation (=1.54056 Å) and *θ* corresponds to the angle of the most intense peak (104). The results are given in Table 2. It can be deduced that the grain size (*D*_SC_) decreases from 55 to 49 nm when we introduce the Ni^2+^ ions.

**Fig. 2:**
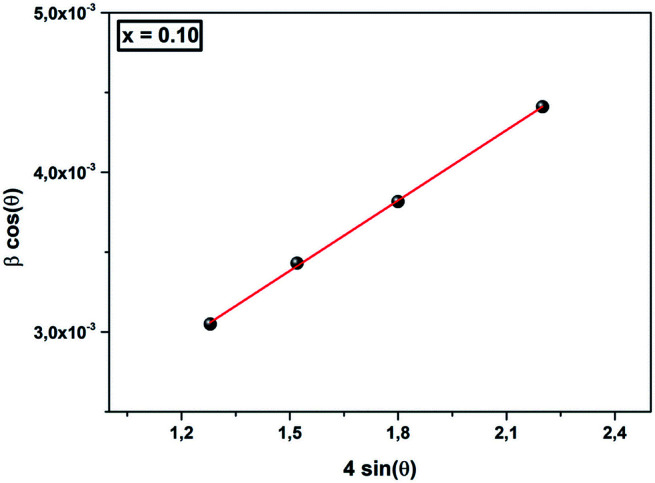
The Williamson–Hall analysis for *x* = 0.10.

As in Scherrer’s method, the crystallite size values were determined from the Williamson–Hall equation:^38^3
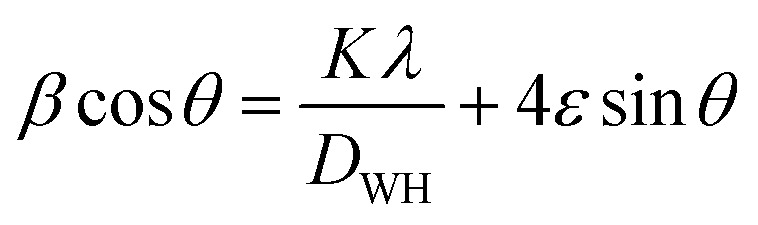
*θ* is the Bragg angle, *ε* is the strain and *D*_WH_ is the crystallite size. The slope of the plot of *β* cos *θ* (*y*-axis) *vs.* 4 sin *θ* (*x*-axis) gives the strain (*ε*) and the crystallite size (*D*_WH_) can be calculated from the intercept of this line on the *y*-axis (Fig. 2). The calculated values are grouped in Table 2. We can deduce from this result that the average crystallite size determined by Williamson–Hall is greater than that obtained by Scherrer’s method, which is due to the broadening effect caused by the strain exhibited in this technique.

### 3.2 Morphological characterization

**Fig. 3:**
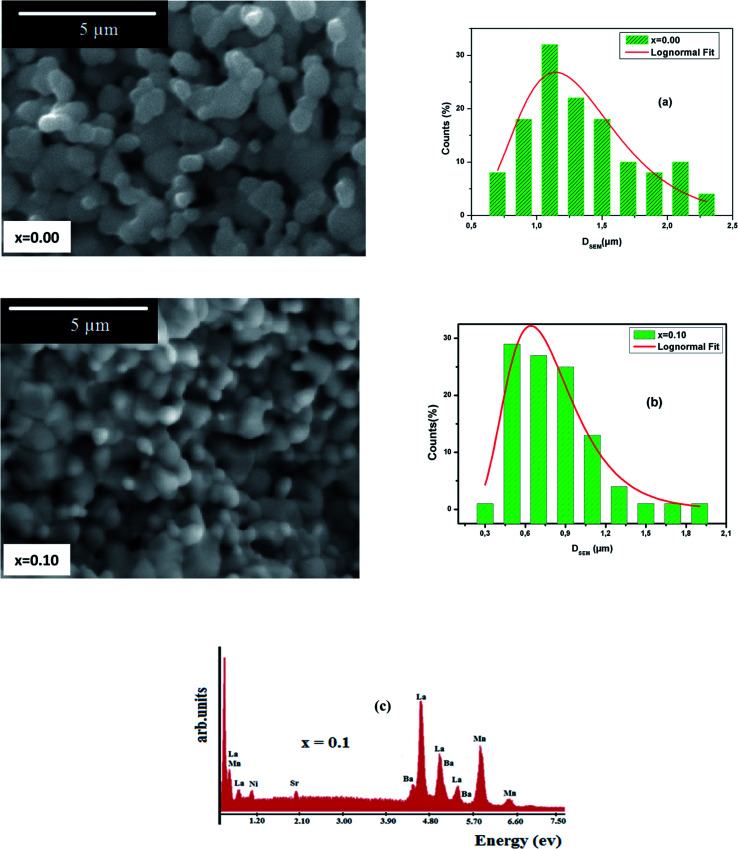
Typical SEM images for *x* = 0.00 and 0.10, (a) and (b) present the histograms of particle size and (c) shows the EDX analysis for *x* = 0.10.

Fig. 3 presents the morphology of La_0.6_Ba_0.2_Sr_0.2_Mn_1−*x*_Ni_*x*_O_3_ (*x* = 0 and 0.1) as examples, demonstrated in the SEM images. It can be seen that the distribution of the grains is uniform over the entire surface and the grains are well joined, which shows that our samples are well formed.

ImageJ software was employed to determine a statistical count of the grain size, which was performed on the SEM images. This technique consists of measuring the diameters of all the particles in the SEM image. Then, we adjusted these data using the log-normal function.4
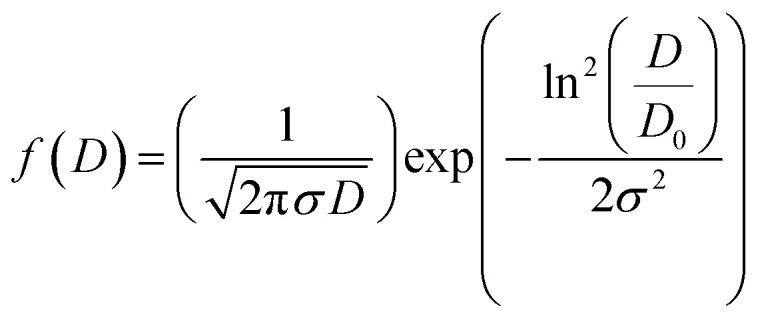


Where, *σ* is the median diameter obtained from the data dispersions and *D*_0_ is the median diameter obtained from the SEM images. Fig. 3(a) and (b) present the grain number (counts) *versus* the particle size. Using the fit results, the mean diameter 
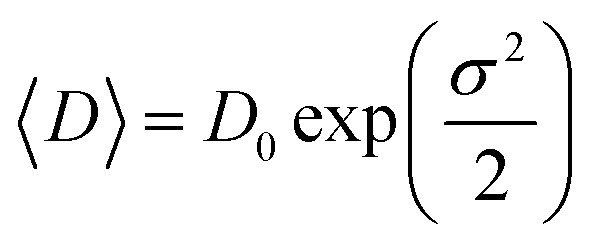
 and standard deviation *σ*_D_ = 〈*D*〉[exp(*σ*^2^) − 1]^1/2^ were determined (Table 2).

It is remarkable that the average size of the particles obtained is greater than the average size of the crystallites determined by XRD. This can be explained by the fact that each particle observed by SEM is made up of several crystallites. The energy dispersive X-ray microanalysis (EDX) spectrum of La_0.6_Ba_0.2_Sr_0.2_Mn_0.9_Ni_0.1_O_3_ is presented in Fig. 3(c) as an example. This technique confirms the composition and purity of the samples. The spectra reveal the homogeneous distribution of La, Ba, Sr, Mn, Ni and O atoms over a wide surface area.

To evaluate the porosity 
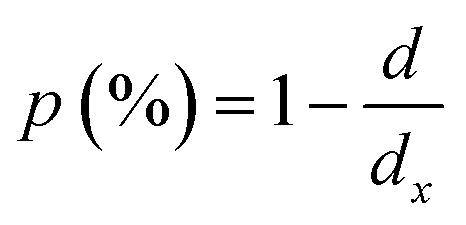
 of the compounds, we calculated the X-ray density, 
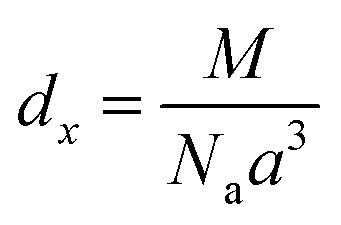
, where *d* is the bulk density, *a* is the lattice constant, *M* is the molecular weight and *N*_a_ is Avogadro’s number^39^ (see Table 2).

### 3.3 Magnetic properties

**Fig. 4:**
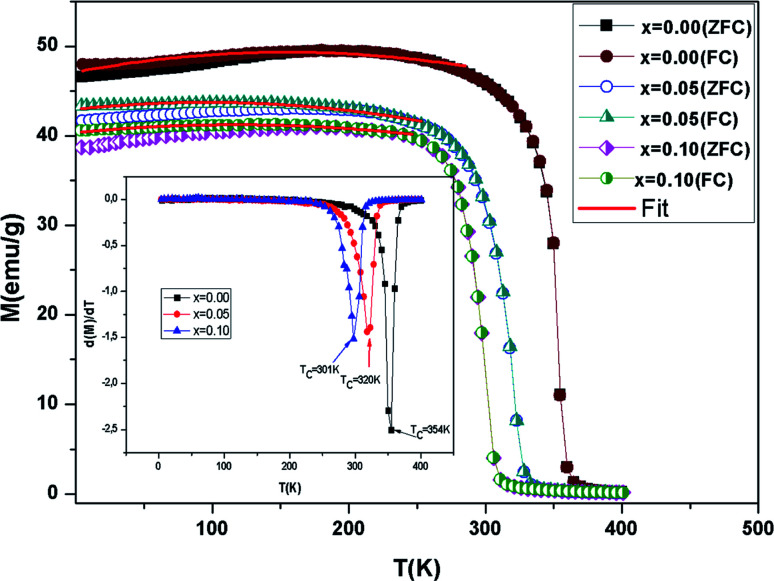
Evolution of magnetization *vs.* temperature under a magnetic field *μ*_0_*H* = 0.05 T for the compounds LBSMNO. The inset: d*M*/d*T* curve *vs. T*.

The evolution of *M*(*T*) measured at 0.05 T in cooled zero field (ZFC) and cooled field (FC) modes is presented in Fig. 4. It is observed that, in the low temperature region, the FC and ZFC curves diverge considerably for all samples, which proves the existence of typical spin glasses, which may also be ascribed to magnetic anisotropy. A spin glass-like state is generally prompted by the coexistence of competing AFM and FM interactions.^40^ With a decreasing temperature, a PM–FM phase transition was observed at the Curie temperature. *T*_C_ is given at the lowest point of the first derivative of the curve *M*(*T*) (d*M*/d*T*) (inset Fig. 4). The *T*_C_ values go from 354 K for *x* = 0.00 to 301 K for *x* = 0.10. This change has been attributed to the modification of the Mn–O–Mn bond angle. Doping with the slightly larger Ni^2+^ (*r*_Ni_^2+^ = 0.69 Å) for Mn^3+^ (*r*_Mn_^3+^ = 0.645 Å) decreases the mean value of the radius of the manganese-site, alters the Mn^3+^/Mn^4+^ ratio and decreases the bond angle *θ*_(Mn/Ni)–O–(Mn/Ni)_. In the La_0.6_Ba_0.2_Sr_0.2_MnO_3_ sample, ferromagnetism is clarified by the DE interaction between the Mn^3+^ and Mn^3+^ ions. Taking into account the neutrality of the charges (La_0.6_^3+^Ba_0.2_^2+^Sr_0.2_^2+^(Mn_0.6−*y*_^3+^Mn_0.4−*x*+*y*_^4+^)Ni_*x*_^2+^O_3_^2−^) of the samples, the substitution of Ni^2+^ transforms the average valence state of Mn^3+^ to Mn^4+^. This weakening of the Mn^3+^ ions essentially induces a reduction in the jumps of the electrons (*e*_g_) and the progressive suppression of the DE interaction subsequently leads to a reduction in ferromagnetism. Moreover, the competition between the AFM and FM interaction exchange is reinforced.^41^ On the other hand, when the Ni content increases, *M* decreases in the FM region and that is consistent with the results in ref. 16 and 42.

The most important cause of the decrease in *T*_C_ is the reduction of the one-electron bandwidth *W*_d_. It is given as:^43^5
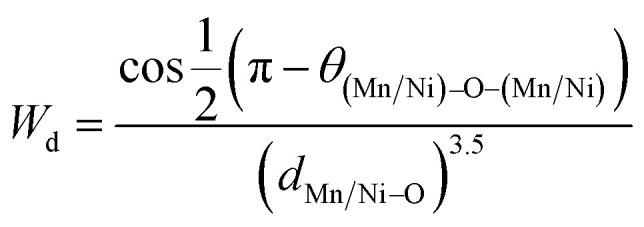


The increase in the Ni content results in a greater length of the *d*_(Mn/Ni)–O_ bond and a reduction in the bond angle *θ*_(Mn/Ni)–O–(Mn/Ni)_, and therefore a weaker bandwidth *W* (Table 2) which causes the decrease of *T*_C_.^44,45^ In fact, this reduction in *W* leads to a reduction in the FM coupling between neighboring manganese atoms.

In addition, it is interesting to understand the behavior of *M vs.* temperature in the FM region. In this context, and according to Lonzarich and Taillefer,^46^ magnetization obeys the theory of spin waves. At low temperatures, this theory is that the magnetization has multiplied in *T*^3/2^ (Bloch’s law) and over a wide range of temperatures in *T*^2^, yet in the vicinity of *T*_C_ it varies as follows: (1 − *T*^4/3^/*T*_C_^4/3^)^1/2^.

In the FM region, the *M* data has been adjusted by this relation:

6 *M*(*T*) = *M*_0_ + *M*_3/2_*T*^3/2^ + *M*_2_*T*^2^

**Fig. 5:**
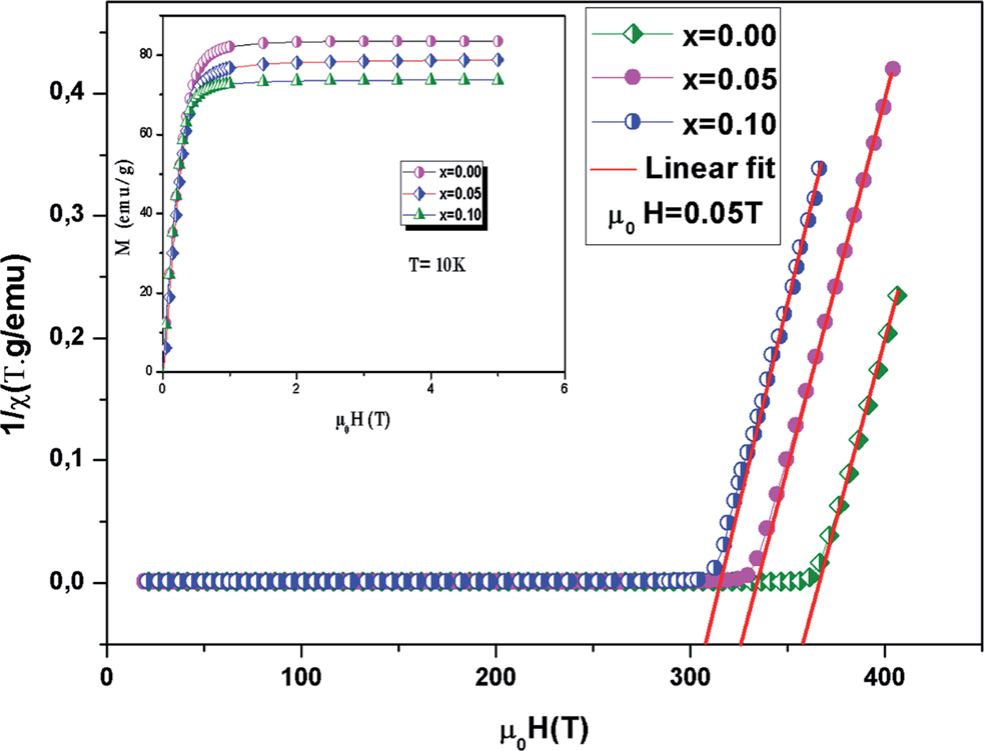
The inverse magnetic susceptibility *χ*^−1^*versus* temperature. The inset shows the field dependence of the magnetization curves at 10 K.

Here *M*_0_ is the spontaneous magnetization. Fig. 5 presents the best fit curves. It can be confirmed that the FM behavior of La_0.6_Ba_0.2_Sr_0.2_Mn_1−*x*_Ni_*x*_O_3_ may be owing to spin waves.

For *T* > *T*_C_, the PM region, the Curie–Weiss law was used to analyse the inverse of magnetic susceptibility (*χ*^−1^ = *H*/*M*):7
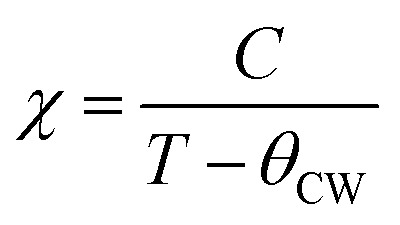
Here, 
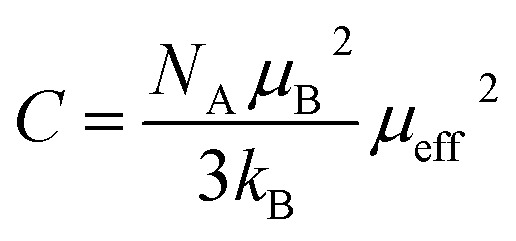
 is the Curie constant and *θ*_CW_ is the paramagnetic-Curie temperature. These parameters were obtained using the fit of curve *χ*_m_^−1^(*T*) (Fig. 5) and its values are also given in Table 3. The positive value of *θ*_CW_ suggests that the ferromagnetic interactions between the nearest neighbors are dominant in the system, which could be due to DE Mn^3+^–O^2−^–Mn^4+^ coupling. When *x* increased, this parameter decreased, which indicates the weakening of the ferromagnetic interactions.^47^ The values of *θ*_CW_ are higher than those of *T*_C_, which indicates the presence of magnetic inhomogeneity above *T*_C_.

**Table 3:** Transition temperature *T*_C_, *θ*_CW_, *μ*^the^_eff_, *μ*^exp^_eff_*vs.* of *x* ratio for LBSMNO

	*x*		
	0.00	0.05	0.10
*T*_C_ (K)	354	320	301
*θ*_CW_ (K)	358	326	308
*μ* ^exp^_eff_ (*μ*_B_)	4.92	4.77	4.64
*μ* ^the^_eff_ (*μ*_B_)	4.48	4.34	4.19
*μ*_0_ *H*_C_ (10^−3^ T)	9.7	8.9	7.7
*M*_s_ (emu g^−1^)	84	79	75
*M*_r_ (emu g^−1^)	13	8	5
*R*	0.066	0.10	0.15

The experimental effective moment *μ*^exp^_eff_ can be calculated using the parameter *C* and the values are given in Table 3.

The theoretical effective paramagnetic moment for La_0.6_Ba_0.2_Sr_0.2_Mn_1−*x*_Ni_*x*_O_3_ compositions could be calculated using the following expression: 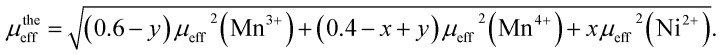


The percentage of Mn^3+^ and Mn^4+^ ions was calculated and checked by the conventional chemical method. The orbital moment is frozen (*L* = 0) for Mn^3+^ and Mn^4+^, so, the theoretical effective moment can be given as: 
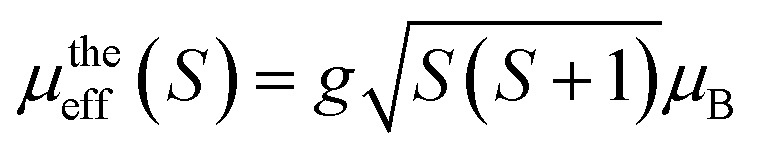
 with *S* = 1.5 for (Mn^4+^, 3d^3^), 2 for (Mn^3+^, 3d^4^) and 4 for (Ni^2+^, 3d^8^) and *g* = 2. The calculated values of *μ*^the^_eff_(Mn^3+^), *μ*^the^_eff_(Mn^4+^) and Ni^2+^ are 4.90 *μ*_B_, 3.78 *μ*_B_ and 2.828 *μ*_B_, respectively. From Table 3, a difference between the experimental and theoretical values of *μ*_eff_ can be observed. This can be clarified by the presence of FM clusters within the PM phase.^48^

The evolution of magnetization *vs. μ*_0_*H* for La_0.6_Ba_0.2_Sr_0.2_Mn_1−*x*_Ni_*x*_O_3_ (0 ≤ *x* ≤ 0.1) at 10 K is depicted in the inset of Fig. 5. At *μ*_0_*H* = 1.5 T, the compounds exhibit a constant value of *M*. The magnetic moments determined by the magnetization data are obtained to be 3.56 *μ*_B_ per formula unit for *x* = 0.00, 3.35 *μ*_B_ per formula unit for *x* = 0.05 and 3.1 *μ*_B_ per formula unit for *x* = 0.1.

The calculated magnetic moment can be determined by: *M*_sp_ = ((0.67 − 2*x*) × 4 + (0.4 + *x*) × 3 + 2*x*) *μ*_B_ = (3.88 − 3*x*) *μ*_B_/f.u.

The magnetic moments of Ni^2+^, Mn^3+^ and Mn^4+^ have 2, 4 and 3 *μ*_B_, respectively. The *M*_sp_ values are 3.88 *μ*_B_ for *x* = 0.00, 3.73 *μ*_B_ for *x* = 0.05 and 3.58 *μ*_B_ for *x* = 0.10. This reduction can be attributed to competition between the ferromagnetic and antiferromagnetic interactions. In addition, the Ni^2+^ ion at the *M* site influences the valence states of the manganese ions, *i.e.* it decreases the Mn^3+^/Mn^4+^ ratio. This proves a reduction in the double Zener exchange (DE), which leads to a decrease in magnetization.

**Fig. 6:**
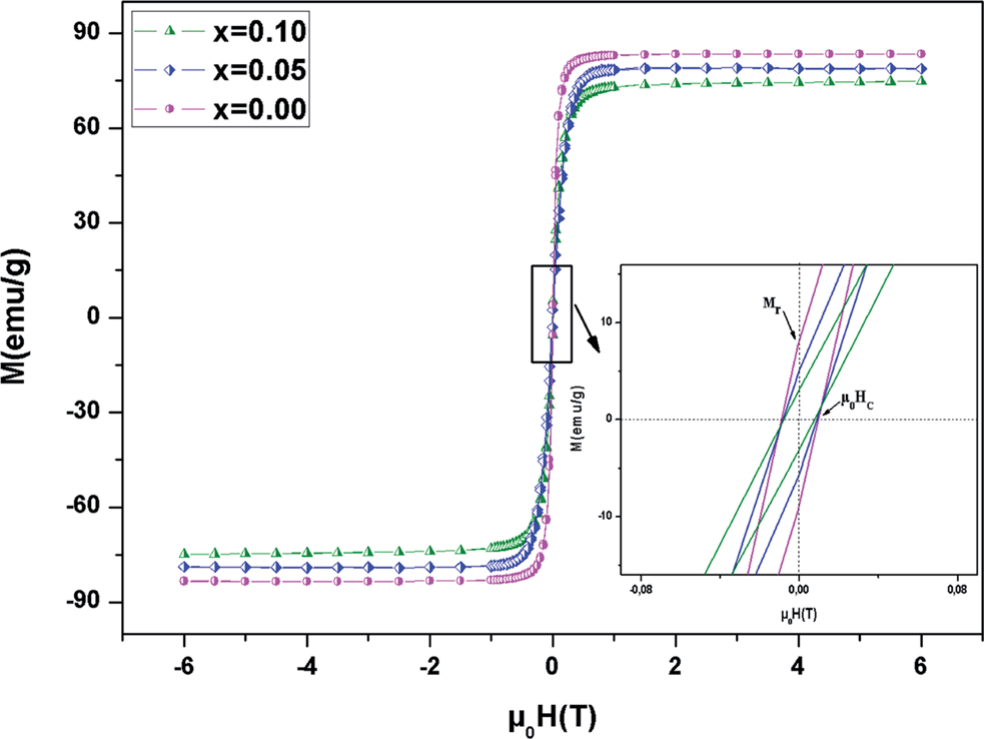
Magnetic hysteresis curves at *T* = 10 K for the LBSMNO samples. The inset shows a zoomed in view of the central portion of the hysteresis at a low field.

We analyzed the hysteresis loops at 10 K (*μ*_0_*H* = ±5 T) to better understand the magnetic properties at low temperatures (Fig. 6). The curves are similar, with hysteresis loops which are weak, with reasonably good coercive fields (*μ*_0_*H*_C_), which decrease with the increase of Ni content . This reduction can be assigned to the decrease in spin dependent electron hopping. In the weak *μ*_0_*H* region, *M* grew significantly and reached saturation as the field increased. The value of *M*_s_ (saturation magnetization) can be estimated at high *μ*_0_*H* at about 5 T. From Table 3, it was found that *M*_s_ decreased, which may be due to antiferromagnetic alignments. Likewise, the insertion of Ni in the Mn site modifies the valence states of the manganese ions and decreases the level of Mn^3+^/Mn^4+^, which in turn weakens the DE interaction. The inset of Fig. 6 shows a zoomed in view of the central portion of *M versus μ*_0_*H*, at small *μ*_0_*H*. The determined values of *μ*_0_*H*_C_ with Ni substitution are summarized in Table 3. The weak hysteresis loop with large saturation values confirms the characteristic soft FM behavior of the compounds. In this context, it can be concluded that our compounds may be applicable to read and write processes in high density recording media or for information storage.^49^

The remanence ratio (*R*) is utilized to comprise the isotropic nature of our investigated compounds. The values of remanence varied in the range of 5–13 emu g^−1^. The ratio (*R*) is given by: *R* = *M*_r_/*M*_s_. We have summarized the values of *R* in Table 3. The small obtained values confirm the isotropic natures.^50^ In this context, for magnetic recording and memory devices,^51^ it is advantageous to have higher remanence ratios. The values of *R* reveal an increasing trend with Ni^2+^ substitution.

### 3.4 Magnetocaloric properties

We have shown previously that there is a phase transition around *T*_C_. Therefore, to calculate the change in magnetic entropy (−Δ*S*_M_), it is necessary to know the order of this transition.

**Fig. 7:**
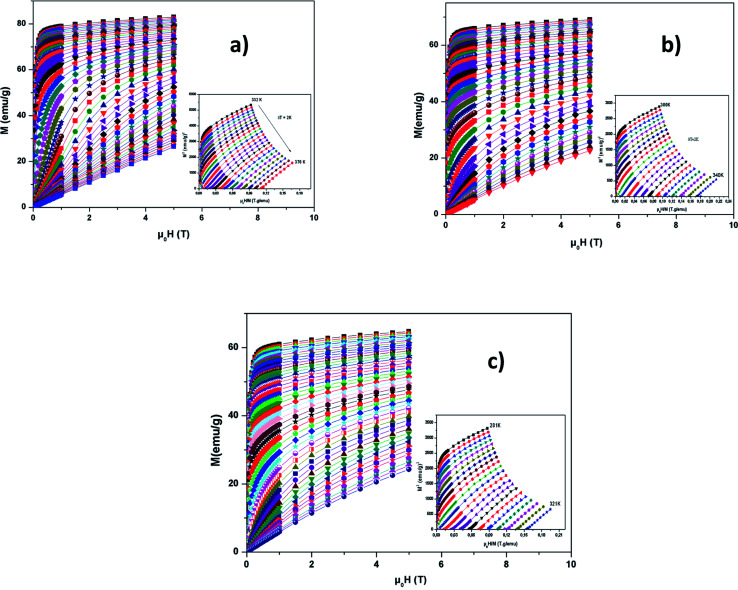
Magnetization *versus* field (*M vs. μ*_0_*H*) curves for LBSMNO compounds at 5 K. The inset: *M*^2^*vs. μ*_0_*H*/*M* plots around *T*_C_.

We have presented in Fig. 7(a–c), the external *μ*_0_*H* variation of the isothermal magnetization at different temperatures around *T*_C_. *M* increases rapidly at low *μ*_0_*H* and then achieves saturation, which shows ferromagnetic behavior. Above *T*_C_, thermally unsettled magnetic moments yield to increase magnetizations linearly at high temperatures, which means PM behavior. This phenomena demonstrates the magnetic phase transition.

In the inset of Fig. 7(a–c), we have presented the Arrott plots *(M*^2^*vs. μ*_0_*H*/*M*) for the La_0.6_Ba_0.2_Sr_0.2_Mn_1−*x*_Ni_*x*_O_3_ (0 ≤ *x* ≤ 0.1) compounds, from these curves we can conclude the nature of the magnetic phase transition. The slope of the curves is positive, so the transition is second order, according to Banerjee’s criteria.^52^ The MCE is an intrinsic characteristic of magnetic materials.^53–55^ Its principle is based on cooling or heating compounds when subjected to a magnetic field under adiabatic conditions, which is maximized when materials are close to their magnetic control temperature.

|Δ*S*_M_| can be determined, using Maxwell’s equations, by the formula:8
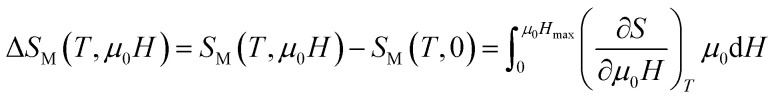
(−Δ*S*_M_) was produced by varying *μ*_0_*H* from zero to *μ*_0_*H* by exploiting Maxwell’s relation
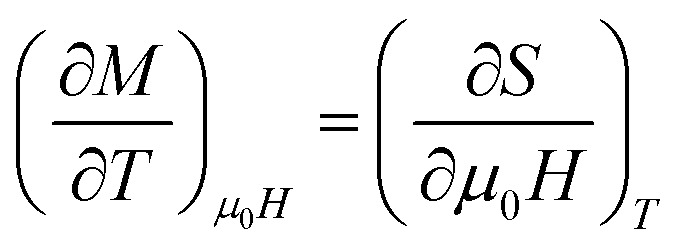


The peaks of the elaborated samples are the same as those of the experiment in the vicinity of *T*_C_ where 
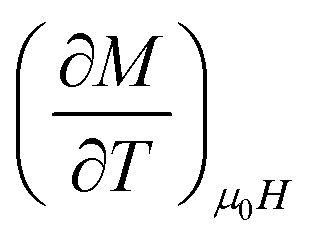
 is the experimental value from the *M*(*T*) curve at *μ*_0_*H*.

We can use the following expression:9
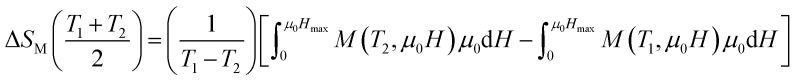
where *T*_1_ represents the temperatures of hot sinks and *T*_2_ represents the temperatures of cold sinks.

**Fig. 8:**
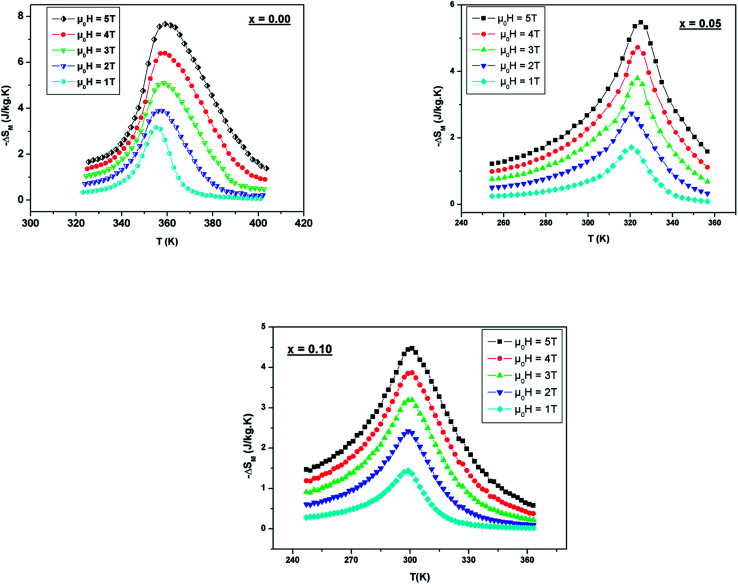
Temperature dependence of the magnetic entropy change under different external fields for LBSMNO manganites.

The predicted (−Δ*S*_M_) *vs.* temperature plots are shown in Fig. 8 at different *μ*_0_*H* values. It can be seen that (−Δ*S*_M_) depends on *μ*_0_*H* and the temperature until a maximum value (−Δ*S*^max^_M_) is reached around *T*_C_. (−Δ*S*_M_) increases with the increase of *μ*_0_*H* for each compound due to the spin effect, which becomes important with the increase of *μ*_0_*H*. The (−Δ*S*^max^_M_) for *x* = 0, 0.05 and 0.1 are 7.65, 5.44 and 4.45 J kg^−1^ K^−1^, respectively, at *μ*_0_*H* = 5 T. Although, when increasing the Ni ratio, (−Δ*S*^max^_M_) and *T*_C_ decrease. This can be explicated by the lowering of the Mn^3+^/Mn^4+^ ratio, which goes from 1.5 (*x* = 0) to 0.8 (*x* = 0.1) and subsequently favors the DE interaction of Mn^3+^–O–Mn^4+^ over the superexchange (SE) interaction of Mn^4+^–O–Mn^4+^, Mn^3+^–O–Mn^3+^ and Ni^2+^–O–Ni^2+^.^56^ This result is strongly affected by structural parameters, such as decreasing the bond angles (Mn/Ni)–O–(Mn/Ni) and increasing the bond distances (Mn/Ni)–O.

(−Δ*S*^max^_M_) is not the only factor that determines the applicability of such a material, but also the temperature range over which it remains considerable is significant.

The relative cooling power (RCP) is another very important parameter along with (−delta), which defines the amount of heat that can be released between cold and hot sinks in an ideal refrigeration cycle and can be given by the following formula:^57^

10 RCP = −Δ*S*^max^_M_ × *δT*_FWHM_

Here Δ*S*^max^_M_ represents the maximum of Δ*S*_M_ and *δT*_FWHM_ is the full width at half maximum.

**Fig. 9:**
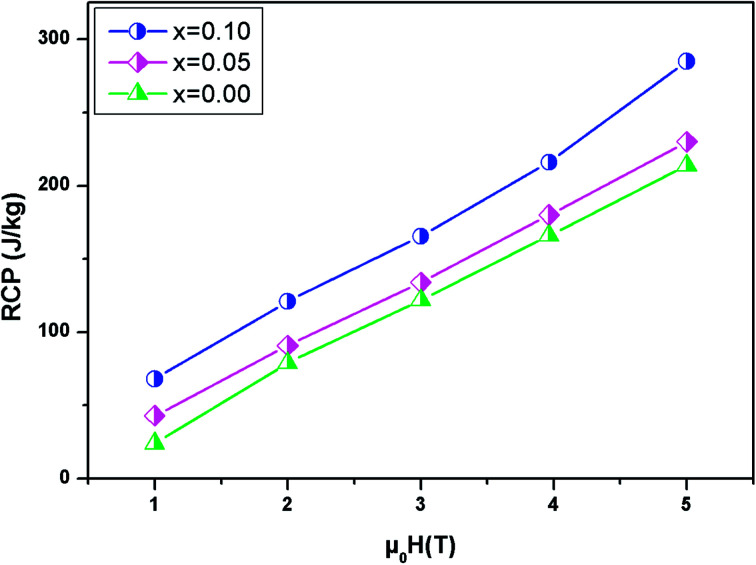
Evolution of RCP *vs.* magnetic field for the LBSMNO compounds.

Fig. 9 present the relative cooling power as a function of *μ*_0_*H* for our compounds. The values of RCP increase with increasing *μ*_0_*H* and reache about 214 J kg^−1^ for *x* = 0, 230 J kg^−1^ for *x* = 0.05 and 285 J kg^−1^ for *x* = 0.1 at *μ*_0_*H* = 5 T. To better understand the performance of the MCE of our compounds, the values of (−Δ*S*^max^_M_) and RCP are compared to other manganites, as given in Table 4.^58–62^ It can be noted that our samples, especially for *x* = 0.1, have a suitable *T*_C_ value, close to RT, and a relatively large magnetic entropy change to include other materials. This proves that our materials can be used in the field of magnetic refrigeration.

**Table 4:** Summary of LBSMNO magnetocaloric values compared to other manganite materials

Composition	*T*_C_	|Δ*S*^max^_M_| (J kg^−1^ K^−1^)	RCP (J kg^−1^)	*μ*_0_ *H* (T)	Ref.
Gd	293	5	153	2	46
Gd	293	9.5	410	5	47
Gd_5_(Sr_2_Ge_2_)	275	18.5	535	5	47
La_0.7_Sr_0.3_Mn_0.95_Ti_0.05_O_3_	308	2.2	90	2	48
La_0.6_Ba_0.2_Sr_02_MnO_3_	354	7.65	214	5	This work
La_0.6_Ba_0.2_Sr_0.2_Mn_0.95_Ni_0.05_O_3_	320	5.44	230	5	This work
La_0.6_Ba_0.2_Sr_0.2_Mn_0.9_Ni_0.1_O_3_	301	4.45	285	5	This work
La_0.6_Ba_0.2_Sr_02_MnO_3_	354	3.88	82	2	This work
La_0.6_Ba_0.2_Sr_0.2_Mn_0.95_Ni_0.05_O_3_	320	2.69	98	2	This work
La_0.6_Ba_0.2_Sr_0.2_Mn_0.9_Ni_0.1_O_3_	301	2.37	121	2	This work
La_0.7_Sr_0.3_Mn_0.9_Fe_0.1_O_3_	260	1.7	83	2	49
La_0.67_Sr_0.33_Mn_0.9_Cr_0.1_O_3_	328	5	—	5	50

On the other hand, to affirm the nature of phase transition, Franco *et al.*^63^ proposed a phenomenological universal curve for the field dependence of Δ*S*_M_. We can construct the universal curve by normalizing all the Δ*S*^max^_M_: Δ*S*_M_(*T*, *μ*_0_*H*)/Δ*S*^max^_M_ below and above *T*_C_, by imposing that the position of two additional reference points in the curve correspond to *θ* = ±1.11
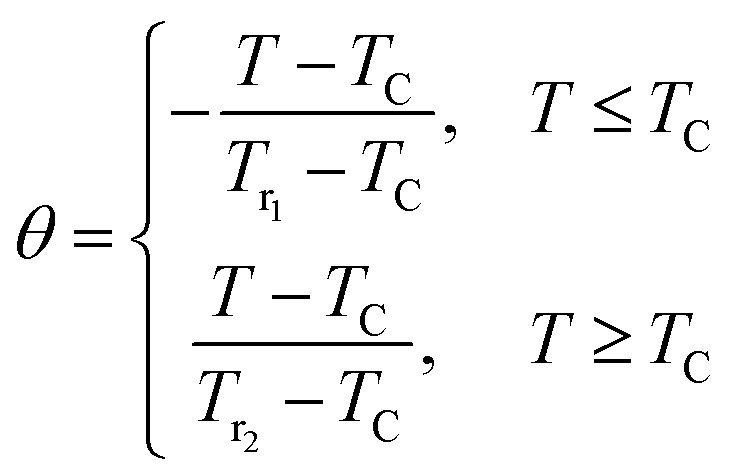
Here *T*_r1_ and *T*_r2_ are chosen as reference temperatures, such that Δ*S*_M_(*T*_r1,2_) = 1/2Δ*S*^max^_M_.

**Fig. 10:**
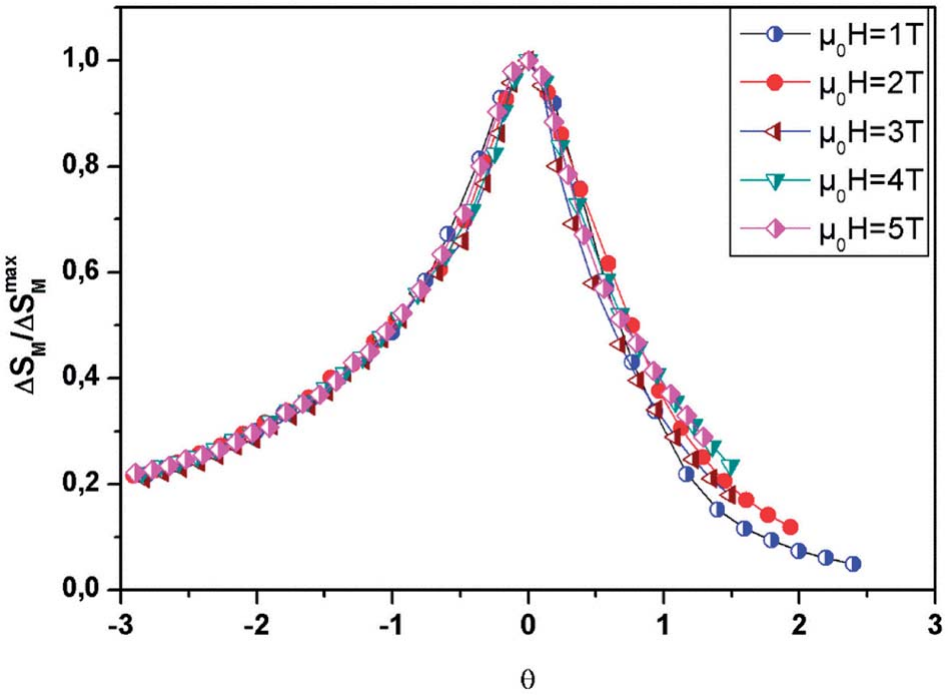
Universal behavior of the scaled Δ*S*_M_ curves of the LBSM_0.95_N_0.05_O sample under various fields.

By referring to Banerjee’s criteria for a 2^nd^ order PM–FM transition, all Δ*S*_M_ curves at different *μ*_0_*H* values should merge into one curve with temperature scaling. If not, the samples follow a 1^st^ order phase transition. Fig. 10 represents the evolution of Δ*S*_M_(*T*, *μ*_0_*H*)/Δ*S*^max^_M_*vs.* temperature *θ* at different *μ*_0_*H* values for *x* = 0.05, for example. In this figure, all the data collapses into a single master curve around *T*_C_, indicating the 2^nd^ order nature of this phase transition. These results are in good accordance with those obtained by the Banerjee criterion discussed before. In addition, this universal curve can be used for practical purposes, such as extrapolating results to fields or temperatures not available in the laboratory, improving data resolution and deconvoluting the response of overlapping magnetic transitions.^64^

We can adjust this curve by the Lorentzian function:12
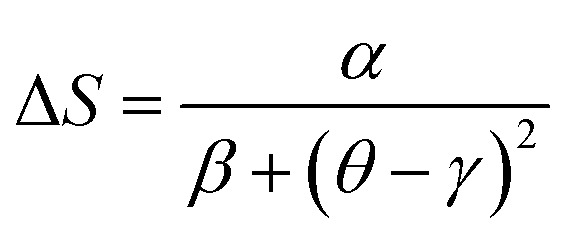
Here *α*, *β* and *γ* are adjusted parameters. Given the asymmetry of the curve, two ensembles of different constants must be used:

• *α* = 1.85 ± 0.03, *β* = 1.55 ± 0.02 and *γ* = 0.53 ± 0.04, for *T* ≤ *T*_C_.

• *α* = 1.16 ± 0.01, *β* = 1.12 ± 0.02 and *γ* = − 0.07 ± 0.03, for *T* > *T*_C_.

From eqn (7), the stance and magnitude of the peak, namely, (Δ*S*^max^_M_, *T*_C_) and *T*_r_1__ and *T*_r_2__ are the only ones that are to describe Δ*S*, where *T*_r_2__ > *T*_C_ and *T*_r_1__ < *T*_C_. In the end, to transpose Δ*S*(*θ*) into the real Δ*S*_M_(*T*), we only use these values, which are fixed by the properties of the compounds.

It is essential to study how the MCE evolves over the ranges of applied magnetic fields and the desired temperatures, taking into account that the 2^nd^ order transition has been proven for all samples.

The evolution of the Δ*S*_M_*vs.* the field is given by the expression, according to Parker and Oesterreicher:^65^ Δ*S*^max^_M_ = *b*(*μ*_0_*H*)^*n*^, *b* is a constant and *n* is an exponent, which depends on the magnetic state of the sample. The exponent *n* can be expressed by the following expression:13
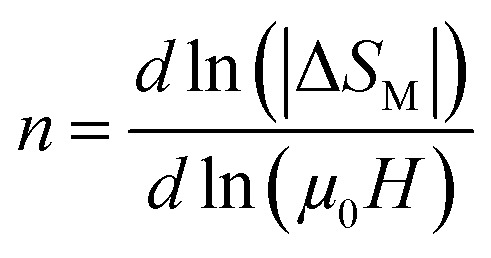


**Fig. 11:**
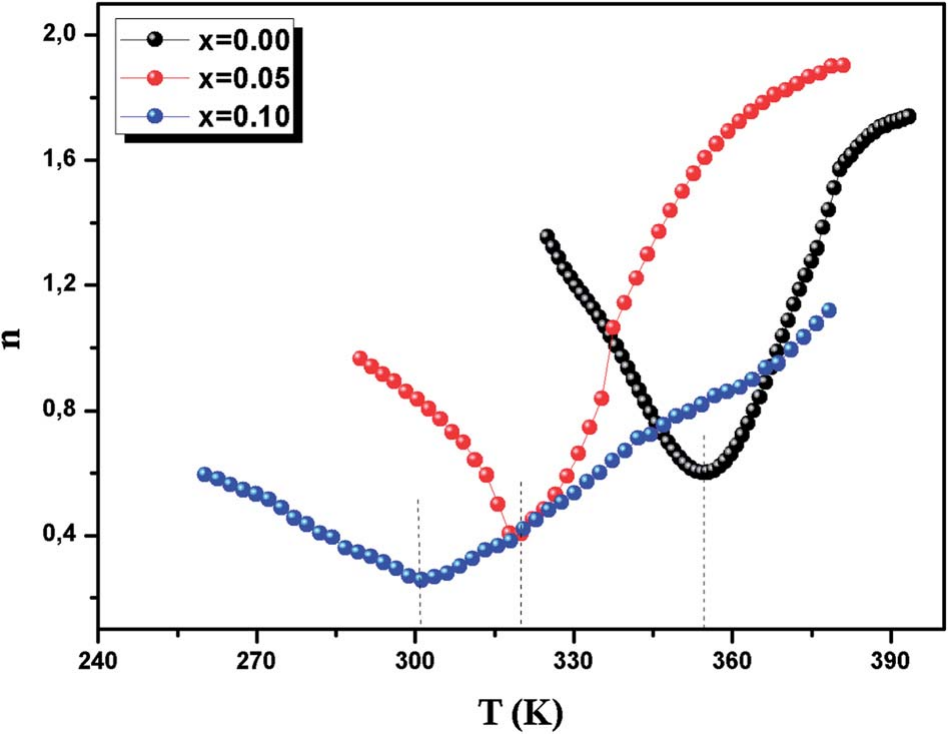
Variation of the exponent *n vs.* temperature for the LBSMNO samples.

According to the mean field approach for conventional ferromagnetic compounds, a minimum value of “*n*” is 2/3 at *T*_C_. Below *T*_C_, *n* has been predicted to be 1 and the materials are in the FM state. However, above *T*_C_, it is equal to 2 in the PM zone, according to the Curie–Weiss law. Yet, recent experimental data indicates a deviation from *n* = 0.66, in the case of a few soft magnetic amorphous compounds. The temperature dependence of *n* is shown in Fig. 11. The values of *n* are found to be 0.67, 0.45 and 0.32 for *x* = 0.00, 0.05 and 0.10, respectively. For *x* = 0, the value is close to the values of the mean field model. However, for *x* = 0.05 and 0.1, these values do not coincide with the predicted value of the mean field of 0.66. This difference is probably due to local inhomogeneities around *T*_C_.^66^

### 3.5 Electrical properties

**Fig. 12:**
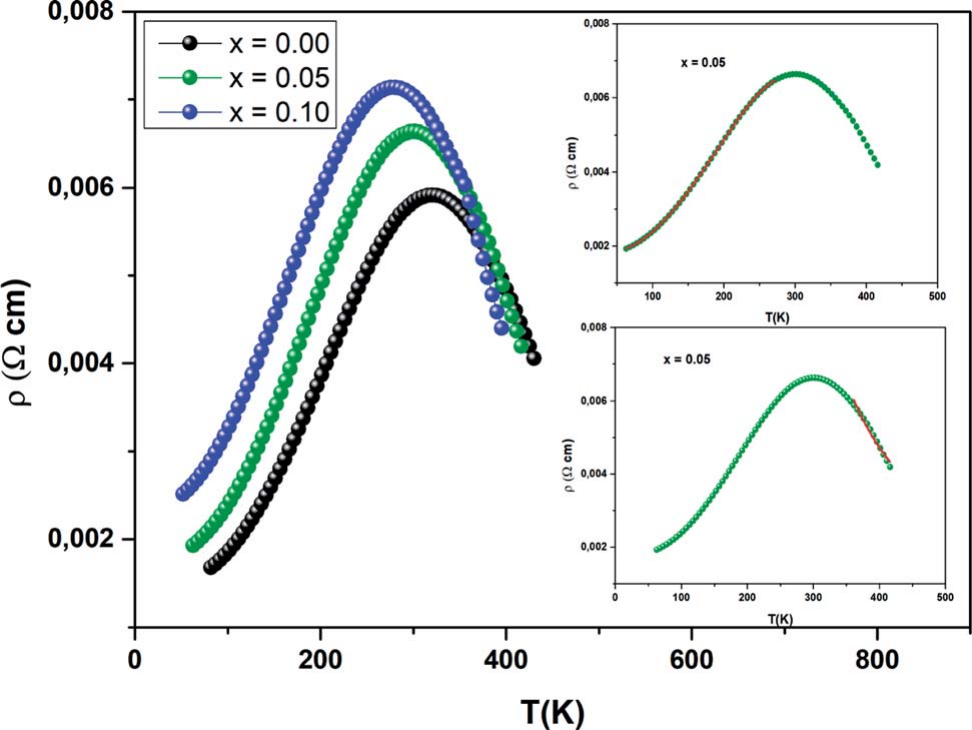
Variation of *ρ vs. T* for three samples. The inset (top) depicts the best fit of the experimental values in the metallic region, for *T* < *T*_M–SC_. The inset (bottom) corresponds to the fit of the data in the semiconducting region (*T*_M–SC_).

Fig. 12 presents the variation of electrical resistivity (*ρ*) *vs.* temperature (*T*) for our samples. All the samples are magnetically ordered and the resistivity exhibits a metallic behavior for low temperatures, resulting from a strong ferromagnetic coupling. Semiconductor behavior (SC) is reported at higher temperatures. It can be concluded that these samples undergo a semiconductor-to-metal (SC–M) transition at *T* = *T*_M–SC_. With increasing Ni concentration, this peak temperature *T*_M–SC_ decreases (Table 5). From this table, one can see that *T*_M–SC_ for all the doped materials is much lower than *T*_C_. From this result, it can be said that the transport properties are governed by the presence of inter-grain boundaries.

**Table 5:** The best fit parameters gated with the experimental resistivity data utilizing eqn (17)

	*x* = 0.00	*x* = 0.05	*x* = 0.10
*ρ*_0_ (Ω cm)	0.0011	0.0015	0.0022
*ρ*_2_ (× 10^−7^ Ω cm K^−2^)	74.39	94.60	1.13
*ρ*_5_ (× 10^−15^ Ω cm K^−5^)	72.73	1.28	2.32
*A* (× 10^−7^ Ω cm)	6.47	4.65	3.45
*E*_a_/*k*_B_ (K)	2100	1390	780
*U*_0_/*k*_B_ (K)	8224	7822	6993
*T* ^mod^_C_ (K)	305	297	275
*R* ^2^	0.9998	0.9995	0.9999

To better understand the contribution of the different factors causing the conduction mechanism below the transition temperature (*T* < *T*_M–SC_), the *ρ*(*T*) curve was fitted using different theoretical models.

Conduction electrons meet different competitors, including scattering of the grain/domain boundary, electron–magnon scattering and electron–electron scattering. Using the following empirical relation,^67^ the electrical resistivity data is analyzed:

14 *ρ*_FM_ = *ρ*_0_ + *ρ*_2_*T*^2^ + *ρ*_5_*T*^5^

Here *ρ*_0_ is residual resistivity due to the domain or grain boundaries, *ρ*_2_*T*^2^ is the contribution from the electron–electron scattering process to the electrical resistivity and *ρ*_5_*T*^5^ is associated with the electron–phonon interaction.^68^

**Fig. 13:**
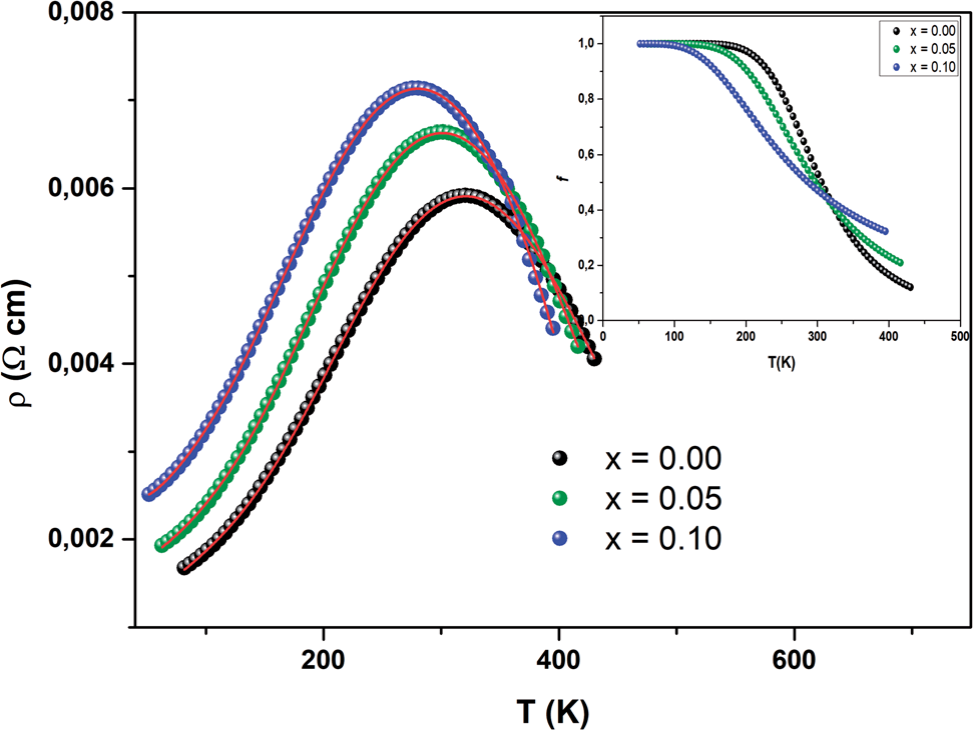
Temperature dependence of resistivity fitted according to eqn (16). The inset shows *f*(*T*) as function of temperature.

The values of the electrical resistivity were adjusted using eqn (14) and in the inset of Fig. 13, we have given the best fit. In Table 5 we have grouped together the estimated values of the adjusted parameters.

Above (*T* > *T*_M–SC_), the resistivity is simulated by the SPH mechanism.^69^

*ρ* is expressed as in the adiabatic SPH model:15
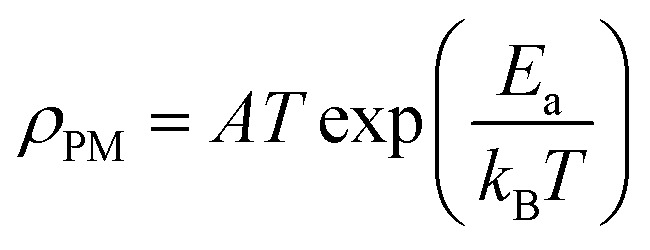
where *A* and *E*_a_ are the coefficient of resistivity and activation energy associated to polaron binding energy, respectively. We have fitted the variation of electrical resistivity (see the inset of Fig. 12) and the results are given in Table 5. We calculated the hopping energies *E*_a_ and we have deduced that the values of *E*_a_ are 180, 86 and 67 meV for *x* = 0, 0.05 and 0.1, respectively.

To understand the transport mechanism of the total resistivity over the whole temperature range, we used a phenomenological percolation model.^70^ For this model, resistivity is defined based on the contributions of the FM clusters in the PM region. Thus, the resistivity is expressed by the following expression:

16 *ρ* = *ρ*_FM_*f* + *ρ*_PM_(1 − *f*)

Here *f* is the volume fraction of the ferromagnetic phase and (1 − *f*) is the volume fraction of the paramagnetic phase.

The volume fraction follows the Boltzmann distribution and this is expressed *via* the following equation:
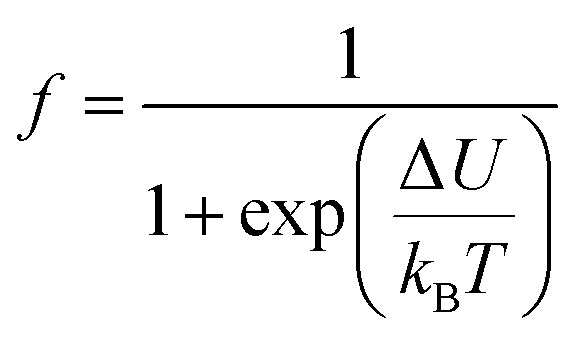
where Δ*U* = −*U*_0_(1 − *T*/*T*^mod^_C_) is the energy gap between the FM and PM states. *T*^mod^_C_ is the temperature of resistivity maxima and *U*_0_ is taken as the energy gap for temperatures lower than *T*^mod^_C_.

With *T* = *T*^mod^_C_, *f* = *f*_c_ = 0.5 where *f*_c_ is called a percolation threshold.^71^ Where *f* < *f*_c_, the sample remains semiconducting and for *f* > *f*_c_ it acquires a metallic phase.^72^

Hence, in the entire temperature range eqn (16), can be given as:17
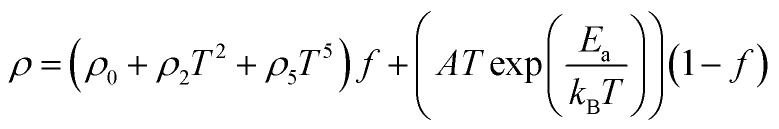


The data evaluated from eqn (17) are in agreement with the experimental results. It can be seen that the percolation model adequately describes the resistivity behavior over a wide range of temperatures, including the phase transition region. We have grouped the most suitable parameters in Table 5 and in Fig. 13, we have presented the fit of the data.

The inset in Fig. 13 shows *f*(*T*) *vs.* temperature for all samples. Below *T*_M–SC_, the volume concentration of the ferromagnetic phase remains equal to 1. This proves the strong dominance of the FM part in this zone. Afterwards, *f*(*T*) starts to lower to zero, since the metallic state (FM) moves to a semiconductor state (PM). This result proves the validity of the percolation approach.

## 4. Conclusion

We have investigated the physical properties of polycrystalline La_0.6_Ba_0.2_Sr_0.2_Mn_1−*x*_Ni_*x*_O_3_ samples. Their crystal structures correspond to a rhombohedral structure, with the *R*3̄*c* space group without any secondary phase. When the substitution rate increases, the unit cell volume decreases. The magnetic and electrical measurement data indicate that our compounds show FMM behaviour at low temperature (*T* < *T*_M–SC_) and PMS behaviour above *T*_M–SC_. This temperature decreases as the Ni substitution increases, due to the bandwidth reduction. The values of (−Δ*S*^max^_M_) at *μ*_0_*H* = 5 T are 7.40 J kg^−1^ K^−1^, 5.6 J kg^−1^ K^−1^ and 4.48 J kg^−1^ K^−1^ for *x* = 0.00, 0.05 and 0.10, respectively. The magnetocaloric performance of these samples indicates that the polycrystalline La_0.6_Ca_0.1_Sr_0.3_Mn_1−*x*_Ni_*x*_O_3_ compounds are good candidates for magnetic refrigeration at room temperature.

## Conflicts of interest

There are no conflicts to declare.
