# Correction: Correction: Influence of Ni content on structural, magnetocaloric and electrical properties in manganite La_0.6_Ba_0.2_Sr_0.2_Mn_1−*x*_Ni_*x*_O_3_ (0 ≤ *x* ≤ 0.1) type perovskites

**DOI:** 10.1039/d4ra90015d

**Published:** 2024-04-08

**Authors:** Ahmed Dhahri, J. Laifi, Soumaya Gouadria, M. Elhadi, E. Dhahri, E. K. Hlil

**Affiliations:** a Department of Physics, College of Science and Humanities – Dawadmi, Shaqra University Riyadh Saudi Arabia dhahridhahri14@gmail.com; b Laboratoire de Physique Appliquée, Faculté des Sciences de Sfax, Université de Sfax Tunisia; c Physics Department, College of Science, Jouf University P.O. Box: 2014 Sakaka Saudi Arabia; d Department of Physics, College of Science, Princess Nourah Bint Abdulrahman University P.O. Box 84428 Riyadh 11671 Saudi Arabia; e Univ. Grenoble Alpes, CNRS, Grenoble INP, Institut Néel 38000 Grenoble France

## Abstract

Correction for ‘Correction: Influence of Ni content on structural, magnetocaloric and electrical properties in manganite La_0.6_Ba_0.2_Sr_0.2_Mn_1−*x*_Ni_*x*_O_3_ (0 ≤ *x* ≤ 0.1) type perovskites’ by Ahmed Dhahri *et al.*, *RSC Adv.*, 2023, **13**, 28158–28158, https://doi.org/10.1039/D3RA90093B.

The authors regret that affiliations *a* & *b* were incorrectly shown in the original manuscript and in the original correction notice. The corrected list of affiliations is as shown here.

The Royal Society of Chemistry apologises for these errors and any consequent inconvenience to authors and readers.

## Supplementary Material

